# Analysis of the association of NPHS2 and ACTN4 genes polymorphism with nephrotic syndrome in Egyptian children

**DOI:** 10.1007/s11033-023-08387-4

**Published:** 2023-04-04

**Authors:** Mohammed F. al-azzawy, Mohammad Al-Haggar, Afaf M. ElSaid, Omali Y. El-khawaga

**Affiliations:** 1 Biochemistry Division, Chemistry Department, Faculty of Science, Mansoura University, Mansoura, 35516 Egypt; 2 Genetic Unit, Department of Pediatrics, Faculty of Medicine, Mansoura University, Mansoura, 35516 Egypt

**Keywords:** NPHS2 gene, ACTN4 gene, Nephrotic syndrome, Single nucleotide polymorphism

## Abstract

**Background:**

One of the most common kidney illnesses in developing countries is pediatric nephrotic syndrome (PNS), which is frequently associated with dyslipidemia and edema. The rapid discovery of genes related to NS has aided in the understanding of the molecular mechanics of glomerular filtration. The goal of this study is to determine the relationship between NPHS2 and ACTN4 in PNS youngsters.

**Methods:**

A study with 100 NS children and 100 healthy matched volunteers was conducted. Genomic DNA was extracted from peripheral blood. Single-nucleotide polymorphisms were genotyped using ARMS-PCR.

**Results:**

A substantial decline in the level of albumin was found in NS cases (*P* < 0.001) Further on, a significantly difference in T.C and TG level between healthy and NS patient. Molecular study showed a highly significant difference of NS patients from controls regarding NPHS2 rs3829795 polymorphic genotypes as the GA heterozygous genotype shows highly significant difference from controls (*P* < 0.001) as well as GA + AA genotypes (*P* < 0.001) in comparison with GG genotype. Regarding rs2274625, The GA heterozygous genotype showed no statistically significant difference between genotypes and alleles with NS (*P* = 0.246). Association of AG haplotype NPHS2 rs3829795–rs2274625 haplotypes found a significant association with the risk of developing NS (*P* = 0.008). Concerning the ACTN4 rs121908415 SNP, there was no link between this mutation and NS children.

**Conclusion:**

The correlation of AG haplotype NPHS2 rs3829795–rs2274625 haplotypes identified a strong association with the likelihood of getting NS, according to our findings. There was no connection found between the ACTN4 rs121908415 SNP and NS children.

## Introduction

Nephrotic syndrome is known as a collection of a group of primary and secondary renal disorders that share common physical glomerular filtration barrier alterations which results in GFB failure in children and a clinical illness characterized with a variety of renal and also extra-renal characteristics such as proteinuria exceeding 3.5 g/dl, edema, hypoalbuminemia, hyperlipidemia, and hypercoagulability [1]. The etiological causes of NS range from primary renal diseases to systemic illnesses with various histopathological presentations [2] It can also cause glomerulosclerosis and renal failure in the absence of therapy response accounting for 85% of all end-stage renal disease (ESRD) [3]***.*** The main cause of the PNS is unknown. It can classified into two major episodes based on the optimal corticosteroid medications, namely steroid-sensitive nephrotic syndrome (SSNS) and steroid-resistant nephrotic syndrome (SRNS) [4, 5]***.*** The quick revelation of genes that relate to NS has assisted in the knowledge of glomerular filtration's molecular mechanics. Because it is almost certainly a kidney disease confirmation of a genetic mutation usually means a lower chance of recurrence after transplantation [6]***.*** Many genes involved in the Slit diaphragms and actin cytoskeleton influence podocyte function***.*** The NPHS2 gene is located on chromosome 1q25-31 so has a coding region of 1149 bp with eight exons. It encodes podocin, a member of the family Stomatin protein with 383 amino acids that is produced by visceral glomerular epithelial (podocytes) [7, 8]***.*** More than 25 polymorphic variations and 100 harmful mutations have been found in the NPHS2 gene, exist among the gene and includes missense, deletion, and nonsense mutations [9, 10]***.*** The ACTN4 gene was mapped to chromosome 19q13.2 and encodes for "α-actinin-4 protein, an actin-bundling protein of the cytoskeleton which is expressed widely throughout the body but enriched in the kidney podocytes [11]***.*** ACTN-4 has an important role in the function of the podocyte cytoskeleton, with several reports investigating that knock-down or/and overexpression of the transgenic model with ACTN4 is accompanied with podocyte alterations and proteinuria [12]***.*** The mutations throughout ACTN-4 gene were acknowledged as autosomal dominant (AD) late-onset that cause focal segmental glomerular sclerosis (FSGS) [11]***.*** From this point of view, the current study was conducted to evaluate the role of NPHS2(rs3829795–rs2274625) haplotype and ACTN4(rs121908415) polymorphisms in primary nephrotic syndrome in Egyptian children. In addition, some Biochemical and clinical parameters such as serum creatinine, bilirubin, TG, TC, and albumin of control subjects, and NS patients were investigated.

## Materials and methods

A case–control comparative study of 100 children with nephrotic syndrome from the Pediatric Nephrology Unit of Mansoura University Children's Hospital (MUCH) from 2021 to 2022 with 100 children apparently healthy chosen as controls. An informed written consent was taken from all caregivers of patients and control volunteers. Confidentiality of the patients was kept by doing code number for each patient. The samples were collected according to the ethical standards of Institutional Research Board (MS.21.10.1722), Faculty of Medicine, Mansoura University. The study excluded any malignant cells, chronic infectious disorders (including the hepatitis B and C viruses), lupus nephritis, or drug-induced membranous glomerulo-nephritis (MGN). All patients in this study underwent a comprehensive clinical examination by the same clinician, which included imaging tests using x-rays and histopathology, In the laboratory, molecular examinations for genetic changes and some biochemical analyses have been done.

### Blood sampling

Five milliliters of blood were collected by vein puncture from all participants. Each collected blood sample was either dispensed into EDTA-tubes for molecular studies or allowed to collect for collection of serum after centrifugation for biochemical parameters measurement. All samples were obtained then stored at -20°c. Prior to the procedure, they have been left to put in room temperature to use.

### DNA extraction and genotyping

Genomic DNA will extract from peripheral blood cells according to (Thermo Fisher) [13] and using DNA purification capture column kit supplied by (Thermo Scientific GeneJET Genomic DNA Purification Kit #K0721). The purified DNA was used immediately in PCR application. Evaluation of NPHS2 (rs3829795), (rs2274625) and ACTN4(rs121908415) were done using amplification refractory mutation system-polymerase chain reaction (PCR) (Table 1).

**Table 1:** Primer pairs were used to screen for NPHS1 rs437168 and NPHS2 rs3829795 mutation by ARMS-PCR:

Mutation	Primer sequence	Size (bp)
NPHS2 rs2274625 G>A	Common F (FO): 5′-CTGTGGATCACTGAGGGGAG-3′	416
	Common R(RO): 5′-CAAGCACGGTTAAGCATAGAAC-3′	
	R (G allele): 5′-ATCCTAATCTTTCAAGGCCAAC-3′	174
	R (A allele): 5′-GGGGAGTTATTAGCATCGGA-3′	283
NPHS2 rs3829795 G>A	Common F (FO): CATCAACATCAGGCATAAGCAT	292
	Common R(RO): ACAAAAGGTCATCGAATTAGGGT	
	R (G allele): CCTTTCTCTCCTCCCTCCG	201
	R (A allele): CCTTTCTCTCCTCCCTCCA	201
ACTN4 rs121908415 A763G	FO: 5′-CAGACCAGAGCTGATTGAGTATGACA-3′	345
	RO: 5′-GCTGCATCTCCTGGATAGTCTTTTG-3′	
	FI: 5′-GAAGGCCATAATGACCTATGGGC-3′	213
	RI: 5′-CTGAAAAGGCATGGTAGAAGCTTGA-3′	182

### Primer-PCR program

The optimization of amplification was performed under the conditions listed in Table 2. The PCR products were electrophoresed on 2.5%, agarose gel that stained with ethidium bromide and visualized under UV light. ARMS-PCR was used for detection of NPHS2 rs3829795 according to Hashemi et al. method [14] and ACTN4 rs121908415 Primers were designed based on gene sequence [15]***.*** Briefly, two tubes were used to determine each variant for every subject. Each tube contained 8 μl of external primers (4 μl of forwarding control (FO) and 4 μl of reverse control (RO)), 4 μl of DNA,4 μl of A allele primer for tube 1 and 4 μl of G allele primer for tube 2 mixed with 16 μl of master mix (COSMO PCR RED Master Mix (W10203001), willow fort). In an Eppendorf Gradients Thermal cycle, PCR was performed. The selected rs2274625 marker was genotyped using tetra-primer ARMS-PCR. This technique is based on the use of two allele-specific inner primers and two outer primers to amplify three different length fragments from template DNA in a single PCR reaction according to the method of Chamgordani et al. [16]***.***

**Table 2:** Optimization of PCR conditions for of NPHS2 rs2274625, NPHS2 rs3829795 and ACTN4 rs121908415 mutation by ARMS-PCR

Variant name	Cycle name	Temperature (°C)	Time	Number of cycles
NPHS2 rs2274625	Initial denaturation	94	5 min	1
	Denaturation	94	40 s	30
	Annealing	59	40 s	
	Extension	72	50 s	
	final extension	72	10 min	1
	Sock	4	∞	1
NPHS2 rs3289795	Initial denaturation	94	5 min	1
	Denaturation	94	30 s	30
	Annealing	59	30 s	
	Extension	72	30 s	
	final extension	72	10 min	1
	Sock	4	∞	1
ACTN4 rs121908415	Initial denaturation	95	5 min	1
	Denaturation	95	35 s	35
	Annealing	60	35 s	
	Extension	72	40 s	
	Final extension	72	5 min	1
	Soak	4	∞	1

### Biochemical parameters measurement

The following tests were done to all participants according to the manufacturer's protocols; serum of Total cholesterol levels (T.C) (BioMed- L.S, Cat. No. CHO10490), Triglyceride level (T.G) (BioMed- L.S, Cat. No. TG117090), Serum albumin (BioMed, Cat, No. ALB100250), Creatinine level (cat. No. CRE106100).

### Statistical analysis

Statistical programmed for Social Science (SPSS) Version 25.0 was used to revise, encode, and tabulate the obtained data. Pearson's Chi-square test was used to examine the genotype distributions of mutations, as well as the frequency of heterozygous and homozygous for each variant, between patients and controls. It was considered significant if the probability (P) score was less than 0.05 [17, 18].

## Results

The current study represents a clinical trial including about 100 nephrotic syndrome patients and 100 controls from Mansoura University Hospital between 2021 and 2022. Hardy Weinberg equation revealed that all studied genotypes in the control group, as well as in NS cases were in HW equilibrium as no significant differences were found between observed and expected counts in each group. The present results show chosen data of all studied parameters in control subjects compared to cases of NS patients. Patients included 69 males representing the majority of cases and 31 females. The patients ‘age ranged with a mean (± SD) of 11.4 (± 3.8) years. The controls were 100 healthy individuals including 65 male and 35 female. The controls` mean age (± SD) was 10.6 (± 4.3) years. Both patients and healthy controls appeared to be appropriately homogenous concerning age (*P* = 0.155) and gender (*P* = 0.547) distribution.

As seen in Table 3 that showed comparison of serum biochemical tests. Total cholesterol and triglyceride levels were tested in healthy control and NS. Importantly, it was found that a substantial decline in the level of albumin in NS cases (*P* < 0.001) Further on, a significantly difference in T.C and TG level between healthy and NS patient. Additionally, there was no significantly difference between studied groups regarding to bilirubin and creatinine levels (*P* = 0.358, and *P* = 0287 respectively).

**Table 3:** Comparison of biochemical parameters among the studied groups

	Control n = 100	NS (n = 100)	Test of significance	*P*1
Serum albumin (g/dL) Mean ± SD	4.5 ± 0.4	2.8 ± 0.6	F = 24.49	**< 0.001**
Bilirubin(mg/dL) Median (range)	0.7 (0.4–1)	0.6 (0.4–1)	H = 1.804	0.358
s. creatinine(mg/dL) Median (range)	0.5 (0.3–0.7)	0.6 (0.3–6)	H = 29.560	0.287
TC (mg/dL) Median (range)	158 (98–230)	410 (133–784)	H = 12.132	**< 0.001**
TG (mg/dL) Median (range)	78 (50–102)	195 (34–608)	H = 12.761	**< 0.001**

Bold values are highly significant

TC, total cholesterol, TG, triglyceride, SD, standard deviation, F, ANOVA coefficient; H, Kruskal Wallis coefficient. *P* = probability, *P* ≤ 0.001 = highly significant, *P* > 0.05 = non-significant, n = number of cases. P1, comparison between control and all NS

Most of studied cases had no abnormality in US (79%), while 15% had type I and 6% had type II nephropathy. A percutaneous kidney biopsy was performed under general anesthesia in children hospital endoscopy theater. Kidney biopsy was taken during evaluation of patient with different presentation. Out of all studied cases, 55 were subjected to biopsy. Biopsy finding indicate that 44% of NS patients had no abnormality, 7% had minimal change nephrotic syndrome (MCNS), 3% had Membranoproliferative glomerulonephritis (MPGN), while only one case 1% had focal segmental nephrotic syndrome FSNS.

### Distribution of (NPHS2) (rs3829795) and (rs2274625) gene polymorphism in Controls compared to nephrotic cases:

**Fig. 1:**
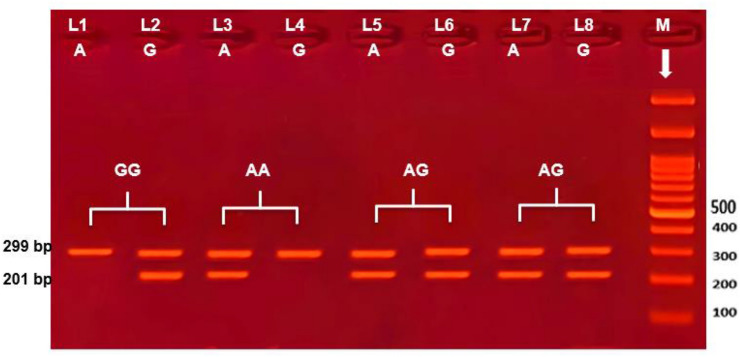
Electrophoretic pattern of NPHS2 rs3829795 by ARMS PCR; M indicate DNA marker (100 BP); 292 bp band shows the internal control. Specific 201 bp bands illustrate G or A alleles based on the primer; Lanes 1 and 2 represent GG homozygous; where G allele appears at lane 2 and A allele absents from lane 1. Lane 3 and 4 represent AA homozygous; where A allele appears at lane3 at 201 bp., G allele absents from lane 4. Lane 5, 6, 7 and 8 represent AG heterozygous; where A allele appears at lane 5, 7. G allele appears at lane 6, 8 at 201 bp

**Fig. 2:**
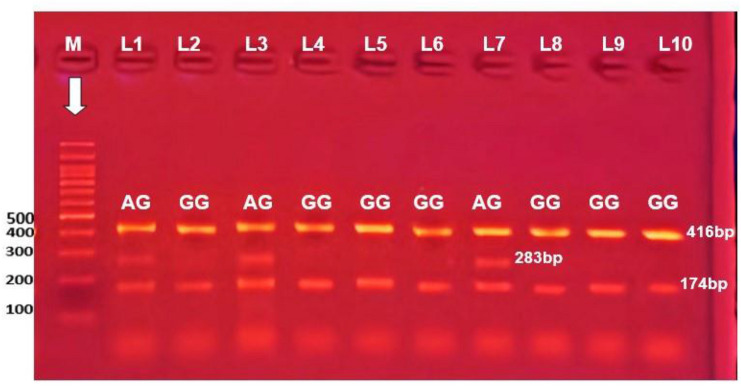
Electrophoretic pattern of NPHS2 rs2274625 by tetra-ARMS PCR. M indicate DNA marker (100 BP); 416 bp band shows the internal control. Specific 283 bp bands illustrate A allele; specific 174 bp bands illustrate G allele. Lanes 2, 4, 5, 6, 8, 9, 10 represent GG homozygous; where G allele appears at 174 bp, A allele absent from them. Lanes 1, 3 and 7 represent AG heterozygous; where both A and G allele appear at 283 bp and 174 bp respectively

Table 4 shows a highly significant difference from controls regarding NPHS2 rs3829795 but not rs2274625 polymorphic genotypes. For (rs3829795), The GA heterozygous genotype was identified in 53 children with NS (53%) higher than in controls (31%). Also, The AA homozygous genotype was higher in NS with 7(7%) children than healthy control children with only 2 (2%). while the GG homozygous genotype was lower in patient (40%) than control (67%).GA genotype shows highly significant difference from controls (odds ratio [OR], 1.927; [CI], 95% (1.338–2.777); (*P* < 0.001)) as well as GA + AA genotypes (OR, 2.002, [CI] 95% (1.402–2.858); (*P* < 0.001)) increased the risk of NS in comparison with GG genotype (wild type). The A allele which represents 67 (33.5%) of NS cases and 35(17.5%) of controls was associated with increased risk of NS (OR, 1.714; [CI] 95%, (1.285–2.278); (*P* < 0.001)) in comparing to G allele (wild type). Figure 1. Regarding rs2274625, The GA heterozygous genotype was identified in 3 children with NS (3%) and not found in controls. Figure 2. Also, The AA homozygous genotype was disappeared in both subjects. While the GG homozygous genotype was lower in patient (97%) than control (100%), no significant association was found between genotypes, alleles with NS (*P* > 0.05).

**Table 4:** Association of rs2274625 and rs3829795 genotypes and alleles with risk of NS susceptibility

NPHS2			Study groups				
			Control (n = 100) %	NS (n = 100) %	OR	(95% confidence interval)	*P*
NPHS2 rs3829795	Genotype	GG	67 (67) %	40 (40%)		Reference	
		GA	31 (31%)	53 (53%)	1.927	(1.338–2.777)	**< 0.001**
		AA	2 (2%)	7 (7%)	2.964	(1.154–7.614)	0.024
		GA + AA	33 (33%)	60 (60%)	2.002	(1.402–2.8587)	**< 0.001**
	Allele	G	165 (82.5%)	133 (66.5%)		Reference	
		A	35 (17.5%)	67 (33.5%)	1.714	(1.285–2.278)	**< 0.001**
NPHS2 rs2274625	Genotype	GG	100 (100) %	97 (97%)		Reference	
		GA	0 (0%)	3 (3%)			0.246
	Allele	G	200 (100%)	197 (98.5%)		Reference	
		A	0 (0%)	3 (1.5%)			0.082

Bold values are highly significant

*P* = probability; *P* ≤ 0.001 = highly significant; OR, odd ratio; CI, 95% confidence interval

### Association of NPHS2 rs3829795- rs2274625 haplotypes among studied groups

The current analyses were performed with data derived from chromosomal region 1q25-31on chromosome 1 with an average distance between SNP markers about 25 kb apart. GG haplotype showed the highest incidence in cases (65.9) as well as in controls (83), while AG haplotype showed the lowest incidence for cases (32.6) and control (17). AG haplotype was significantly associated with risk to develop NS (*P* = 0.008, OR = 1.744, 95% CI (1.156–2.631)).

### Association of NPHS2 rs3829795, NPHS2 rs2274625 with studied parameters

Biochemical parameters including serum, albumin, creatinine, bilirubin, TC, and TG, Also, ultra sound and biopsy finding of all NS patients were investigated with NPHS2 genotypes. Table 5 show no significant Association of studied parameters was found with NPHS2 rs3829795 or rs2274625 among NS patients**.**

**Table 5:** Association of studied parameters with NPHS2 rs3829795 and rs2274625 genotypes among NS patients

	NPHS2 rs3829795					NPHS2 rs2274625								
	NS (n = 100)			Test of significance	*P*	NS(n = 100)		Test of significance	*P*					
	GG(n = 40)	GA (n = 53)	AA (n = 7)			GG(n = 97)	GA(n = 3)							
Serum albumin (g/dL) Mean ± SD	2.8 ± 0.7	2.8 ± 0.6	2.8 ± 0.4	F = 0.130	0.878	2.8 ± 0.6	2.9 ± 0.1	0.118	0.732					
Serum creatinine (mg/dL) Median (range)	0.6 (0.3–6)	0.6 (0.3–5)	0.7 (0.5–3.5)	H = 3.004	0.223	0.6 (0.3–6)	0.6 (0.3–3.5)	0.009	0.925					
Bilirubin (mg/dL) median (range)	0.55 (0.4–1)	0.6 (0.4–1)	0.9 (0.4–1)	H = 3.535	0.171	0.6 (0.4–1)	0.5 (0.5–0.9)	0.175	0.676					
TC (mg/dL) Median (range)	410 (133–629)	410 (236–784)	410 (216–410)	H = 2.872	0.238	410 (133–784)	410 (216–410)	2.152	0.142					
TG (mg/dL) Median (range)	172 (80–436)	219 (34–608)	169 (136–405)	H = 0.733	0.693	205 (34–608)	178 (160–405)	0.134	0.715					
Ultra sound														
Normal	31	77.5%	43	81.1%	5	71.4%	5.361	0.069	78	80.4%	1	33.3%	5.361	0.069
Type 1 nephropathy	8	20.0%	7	13.2%	0	0.0%			14	14.4%	1	33.3%		
Type II nephropathy	1	2.5%	3	5.7%	2	28.6%			5	5.2%	1	33.3%		
Biopsy														
No abnormality	17	85.0%	24	82.8%	3	100.0%	6.501	0.090	43	44.3%	1	33.3%	6.501	0.090
MCNS	2	10.0%	4	13.8%	1	33.3%			6	6.2%	1	33.3%		
MPGN	1	5.0%	1	3.4%	1	33.3%			2	2.1%	1	33.3%		
FSNS	0	0.0%	0	0.0%	1	33.3%			1	1.0%	0	0.0%		

TC, total cholesterol, TG, triglyceride, SD, standard deviation, *P* = probability, *P* > 0.05 = non-significant, n = number of cases. P1, comparison NPHS2 rs3829795 genotypes among NS patients

### Association of ACTN4 rs121908415 genotype and alleles with risk of NS susceptibility:

**Fig. 3:**
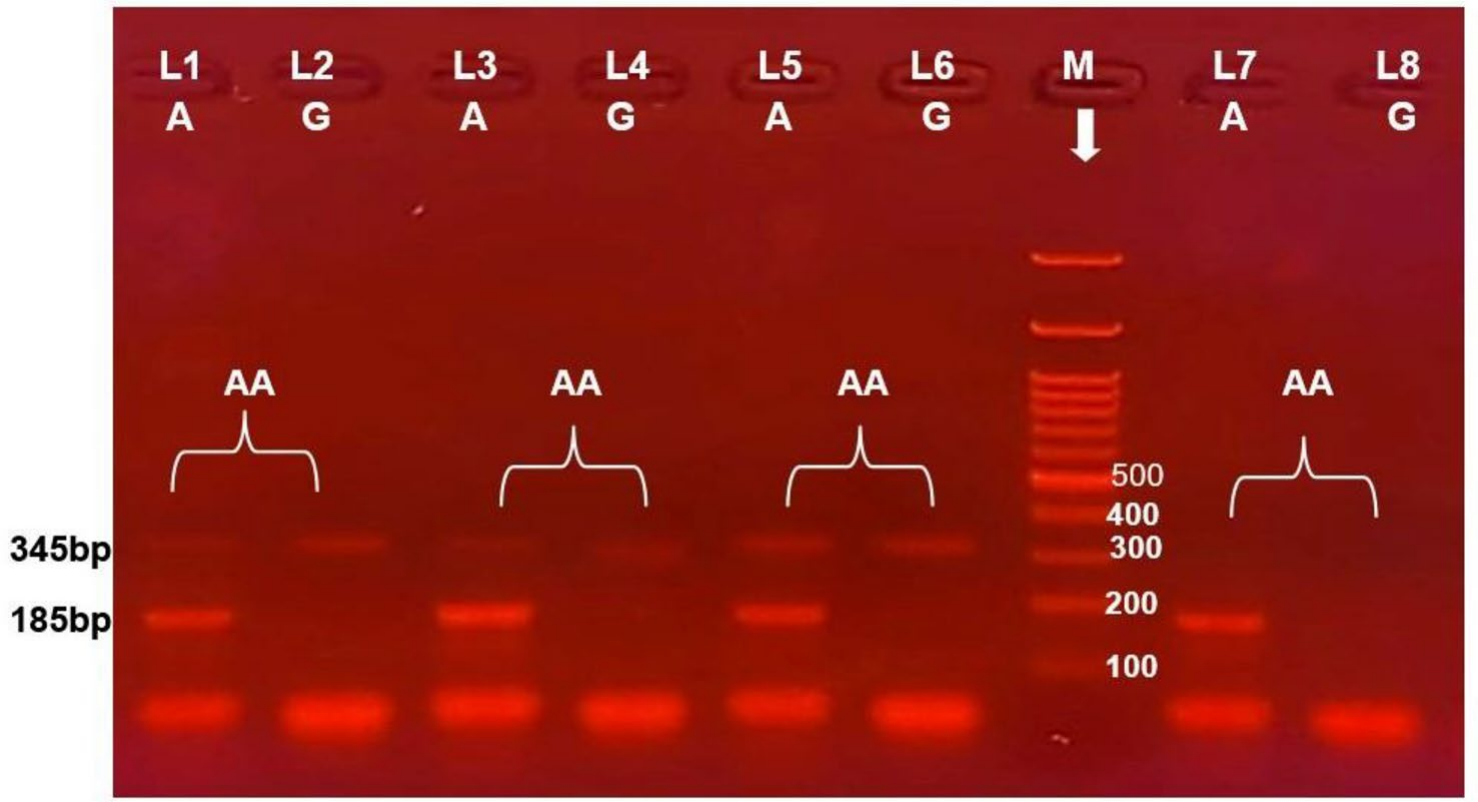
Electrophoretic pattern of ACTN4 rs121908415 by ARMS PCR; M indicate DNA marker (100 BP); 345 bp band shows the internal control. Specific 185,215 bp bands illustrate A alleles based on the primer; Lanes 1, 2, 3, 4, 5, 6, 7 and 8 represent AA homozygous; where lane1, 3, 5, and 7 represents A allele, G allele absent from lane 2, 4, 6 and 8

Regarding ACTN4 rs121908415 SNP, the overall genotype frequencies were 100% for AA genotype, neither AG nor GG genotype was detected in the NS patients and the healthy controls. And there is no difference between studied cases and controls related to AA genotype and A allele. Figure 3.

## Discussion

One of the most frequent kidney disorders in developing nations is a primary nephrotic syndrome (PNS) which is described as NS without systemic illness and has a reported prevalence of 1.5 per 100,000 children each year [19, 20]***.*** NS is mainly characterized by proteinuria > 3.5 g/24 h, oedema, hypoalbuminemia and hyperlipidemia [2]***.*** Proteinuria is the most significant pathophysiologic alteration for PNS, which causes hypoproteinemia and hyperlipidemia (HLP) [21]***.*** It is well known that any factor that destroys the molecular or electrostatic barrier of the glomerular filter could result in proteins from the blood escaping into the urine, which is a significant trigger at the onset of lipid abnormalities. The abnormalities in serum lipoproteins and lipids in nephrotic syndrome are predominantly due to impaired clearance and, to a lesser extent, altered biosynthesis of them by inducing hepatic LDL-receptor gene expression and function, causing hypercholesterolemia [22, 23]. On comparing all biochemical and clinical data between healthy controls and PNS patients using ANOVA, there were no significant differences in serum creatinine, and serum bilirubin between the studied groups. On the other hand, Cholesterol has been found statistically significantly higher in NS (410 mg/dl) compared to control group (158 mg/dl). Also, TGs is statistically significantly higher in NS (195 mg/dl) compared to control group (78 mg/dl). These results are consistent to Yang et al. [24] study as they reported that cholesterol in children with NS plays an important role in the progression of the disease. Also, our finding supported by Hilmanto et al. [25] who reported that there were highly significant differences in those parameters between PNS patients and healthy control in a systematic review to analyze NS complications in children from Asia, Europe, and the US, and Africa.

There are four forms of kidney disorders which can cause pediatric PNS and adolescents. The most prevalent cause of NS in young children is minimal change disease, which can cause very little change to the glomeruli or adjacent structures kidney tissue, Focal segmental glomerulosclerosis which disease can cause some of the kidney's glomeruli to become scarred. Membranous nephropathy (MN) is an autoimmune illness that causes immune proteins to accumulate in the glomerular basement membrane of the kidney. As a result, the membrane thickens and fails to function effectively, enabling too much protein to enter into the urine [26]. Out of all studied cases, 55 were subjected to biopsy, 44 had no abnormality, 7 had MCD representing the most common pathology in NS patients, then 3 cases had MPGN represent the second type, and finally, FSGS which appear on the only case.

Regarding to the etiology of NS in children, epidemiological studies have revealed many environmental factors, inherited, and epigenetic variables are known to be linked to the beginning and progression of the nephrotic syndrome [27]***.*** Nowadays, advances in genotyping techniques have enabled researchers to conduct extensive genome association studies, allowing them to investigate millions of SNPs spread over an individual's whole genome [28]***.***The discovery of genetic abnormalities in the PNS increased the understanding of the molecular basis of NS problems and brought the step closer to discovering a treatment.

The markers of NPHS2 might be applied as biomarker for NS cause several polymorphisms have been discovered assosciated with this disease [29]. Due to more accuracy of haplotype analysis for genotype–phenotype correlations than individual SNPs, it is useful to perform such procedure [30]***.*** Moreover, it is more informative to analyze the biomarkers in groups though each of them could be assessed separately. Also, haplotype analysing could be more informative compared with analyses of individual markers independently, while multiple markers in a unique chromosome were applied to find their relationship with a disease [31]***.***

Among the existing biomarkers in the NPHS2 gene, one intronic marker, rs2274625, and another marker in the promoter region, rs3829795, were explored in this work. In NS instances, the allele and heterozygosity degree of such markers were calculated and compared to healthy participants. For the varient of NPHS2 rs3829795, the NS group had an overrepresentation of the GA and AA genotypes with 53% and 7%, respectively, and a low presentation of GG (40%) compared to healthy children who had a high presentation for GG (67%) and a low frequency of GA (31%) and AA (2%). NPHS2 G>A has a statiscal connection with nephrotic syndrome, indicating that the G allele is the codominant allele in a healthy individual while the A allele increases the risk of nephrotic syndrome. In light of the novelty of our findings, a study was conducted with the same subjects for the rs2274625 variant, which revealed that the G allele had the highest allele frequency with 98.5% frequency and the A allele had the lowest frequency of 1.5% for NS patients compared with healthy children with a high presentation for G allele (100%) and no presentation of A allele. As *P* > 0.05, there is no relationship between NPHS2 rs2274652 and nephrotic syndrome.Interestingly, the current analyses with data derived from chromosomal region 1q25-31 on chromosome 1 with an average distance between SNP markers about 25 kb apart. We also revealed that the frequencies of GG genotype at the SNPs rs3829795 site, rs2274625 site, and haplotype GG in the NPHS2 gene, were less frequent in NS cases, likely signifying that they were protective variants in the disease condition of NS. Moreover, the GA haplotype showed the lowest incidence. GA haplotype was significantly associated with the risk to develop NS (*P* value = 0.008).

These observations are consistent with the findings of Iranian research, which discovered that the G>A allele may be utilized as single-nucleotide markers in linkage analysis to show NPHS2 gene abnormalities in the disease's molecular diagnosis [32]. In a similar study for the northern Chinese population, their data demonstrated that variants in NPHS2 gene were linked to the genetic susceptibility to NS including rs3829795 [33]***.*** We do not know the real impact of these mutations on gene progression but on other study as Caridi et al. [34] found downregulation of NPHS2 gene expression at three variations in the NPHS2 gene promoter region, suggesting that promoter region variants are responsible for regulating NPHS2 gene expression. NPHS2 mutations were reported in patients from Italy, France, and Germany however Japanese children were found not to carry the mutations [35]***.*** The interethnic differences might have a role in the incidence of such mutations.

Zhu et al. [33] present a previous study evaluated a relationship between variants in the NPHS2 gene and proteinuria in PNSin Chinese population. In the present study, no significant difference between biochemical parameters in different genotypes being found among nephrotic patients with G670A rs3829795, rs2274625 genotype, and so on US and biopsy findings.

According to previous studies, the symptoms linked with single mutations in ACTN4 gene in humans and animals involve both gain-of-function and loss-of-function processes. Mutations in ACTN4 in humans cause a family type of FSGS of juvenile or adult onset with an autosomal dominant inheritance (AD) pattern. Also, disease-related mutations increase actinbinding activity in vitro while diverting it from its natural localization in vivo [36]. Patients and healthy subjects in this study were screened for ACTN4 rs121908415 mutation, however no causative ACTN4 was detected as the overall genotype frequencies were 100% of AA genotype. Neither the cases nor the controls were of GG or AG genotype.This suggests that ACTN4 rs121908415 mutation is not a major cause of NS in Egyptian children. Using a greater number of samples may reveal a different possibility in the findings of this gene's connection with nephrotic syndrome in Egyptian children. A previous study evaluated 374 South Indian participants for the existence of this mutation; their classification indicated that the A763G mutation was identified only in three NS patients and not in the controls [15].

In conclusion, The diagnosis of mutations that cause NS is essential for therapeutic considerations and genetic guidance. Although, rs3829795 variant indicate a substantial difference in the polymorphism, and rs2274625 did not show any significance between NS and control, The study's findings in the association of rs3829795 and rs2274625 haplotypes among the groups studied investigated their correlations in Egyptian children which could be employed as biomarkers in genetic analysis to aid in disease diagnosis. Concerning the ACTN4 rs121908415 SNP, there was no association between this mutation and NS children. Upcoming research utilizing larger sample size and excluding some confounding factors is a promising demand.

## Data Availability

The data that supports this work is available upon reasonable request.

## References

[1] Butt L (2020). A molecular mechanism explaining albuminuria in kidney disease. Nat Metab.

[2] De-Seigneux P-Y, Martin S (2009). Management of patients with nephrotic syndrome. Swiss Med Wkly.

[3] Tharaux P-L, Huber TB (2012). How many ways can a podocyte die?. Semin Nephrol.

[4] Webb NJA (2019). Sixteen-week versus standard eight-week prednisolone therapy for childhood nephrotic syndrome: the PREDNOS RCT. Health Technol Assess (Winchester, England).

[5] Hodson EM, Craig JC (2014). Rituximab for childhood-onset nephrotic syndrome. Lancet (London, England).

[6] Wang F (2017). Spectrum of mutations in Chinese children with steroid-resistant nephrotic syndrome. Pediatr Nephrol.

[7] Franceschini N, North KE, Kopp JB, Mckenzie L, Winkler C (2006). NPHS2 gene, nephrotic syndrome and focal segmental glomerulosclerosis: a HuGE review. Genet Med.

[8] McKenzie LM (2007). NPHS2 variation in sporadic focal segmental glomerulosclerosis. J Am Soc Nephrol.

[9] Caridi G, Perfumo F, Ghiggeri GM (2005). NPHS2 (Podocin) mutations in nephrotic syndrome. Clinical spectrum and fine mechanisms. Pediatr Res.

[10] Baylarov R, Baylarova R, Berdeli A, Bayramov R, Haziyev E (2019). NPHS2 gene sequencing results in children of the Azerbaijani population with different types of nephrotic syndrome caused by chronic glomerulonephritis. Bratisl Lek Listy.

[11] Gaber A, Alharthi AA, Hassan MM, El-hallous EI (2020). Screening of ACTN4 and PLCE1 genes mutations in Saudi children patients with steroid resistant nephrotic syndrome. Pak J Biol Sci ISSN.

[12] Kos CH (2003). Mice deficient in α-actinin-4 have severe glomerular disease. J Clin Investig.

[13] Thermo Fisher (2016) Thermo Scientific GeneJET genomic DNA purification Kit #K0721, #K0722, vol 2016, pp 6–13

[14] Hashemi M (2015). Association between NPHS1 and NPHS2 gene variants nephrotic syndrome in children. Iran J Kidney Dis.

[15] Fatima-Jaffer AT, Bhushan-Raju S (2016). α-Actinin-4 gene mutations, steroid responsiveness and FSGS in adult onset-nephrotic syndrome. Hered Genet.

[16] Chamgordani LE, Ebrahimi N, Amirmahani F, Vallian S (2020). CG/CA genotypes represent novel markers in the NPHS2 gene region associated with nephrotic syndrome. J Genet.

[17] Zhao F, Song M, Wang Y, Wang W (2016). Genetic model. J Cell Mol Med.

[18] Kwok P-Y (2000). Approaches to allele frequency determination. Pharmacogenomics.

[19] Dao M (2022). FC067: long-term outcome of childhood onset idiopathic nephrotic syndrome. Nephrol Dialysis Transplant.

[20] Gebrehiwot M, Kassa M, Gebrehiwot H, Sibhat M (2020). Time to relapse and its predictors among children with nephrotic syndrome in comprehensive specialized hospitals, Tigray, Ethiopia, 2019. Int J Pediatr.

[21] Hu P, Lu L, Hu B, Du PF (2009). Characteristics of lipid metabolism under different urinary protein excretion in children with primary nephrotic syndrome. Scand J Clin Lab Investig.

[22] Shearer GC, Stevenson FT, Atkinson DN, Jones H, Staprans I, Kaysen GA (2001). Hypoalbuminemia and proteinuria contribute separately to reduced lipoprotein catabolism in the nephrotic syndrome. Kidney Int.

[23] Rashighi M, Harris JE (2017). 乳鼠心肌提取 HHS public access. Physiol Behav.

[24] Yang F (2014). Association of endothelin-1 gene polymorphisms with the clinical phenotype in primary nephrotic syndrome of children. Life Sci.

[25] Hilmanto D, Mawardi F, Lestari AS, Widiasta A (2022). Disease-associated systemic complications in childhood nephrotic syndrome: a systematic review. Int J Nephrol Renovasc Dis.

[26] Andolino TP, Reid-Adam J (2015). Nephrotic syndrome. Pediatr Rev.

[27] Hussain N (2013). The rationale and design of Insight into Nephrotic Syndrome: Investigating Genes, Health and Therapeutics (INSIGHT): a prospective cohort study of childhood nephrotic syndrome. BMC Nephrol.

[28] Das S, Abecasis GR, Browning BL (2018). Genotype imputation from large reference panels. Annu Rev Genomics Hum Genet.

[29] Joshi BB (2017). Characterization of NPHS2 gene polymorphisms associated to steroid resistance nephrotic syndrome in Indian children. Gene.

[30] Yang J, Cai L, Huang H, Liu B, Wu Q (2012). Genetic variations and haplotype diversity of the UGT1 gene cluster in the Chinese population. PLoS ONE.

[31] Crawford DC, Nickerson DA (2005). Definition and clinical importance of haplotypes. Annu Rev Med.

[32] Chamgordani LE, Ebrahimi N, Amirmahani F, Vallian S (2020). CG/CA genotypes represent novel markers in the NPHS2 gene region associated with nephrotic syndrome. J Genet.

[33] Zhu LI (2009). Genetic effect of the NPHS2 gene variants on proteinuria in minimal change disease and immunoglobulin A nephropathy. Nephrology.

[34] Caridi G (2004). Infantile steroid-resistant nephrotic syndrome associated with double homozygous mutations of podocin. Am J Kidney Dis.

[35] Sako M (2005). Analysis of NPHS1, NPHS2, ACTN4, and WT1 in Japanese patients with congenital nephrotic syndrome. Kidney Int.

[36] Wiggins R-C (2007). The spectrum of podocytopathies: a unifying view of glomerular diseases. Kidney Int.

